# Identification of novel GLI1 target genes and regulatory circuits in human cancer cells

**DOI:** 10.1002/1878-0261.12366

**Published:** 2018-08-30

**Authors:** Yumei Diao, Mohammed Ferdous‐Ur Rahman, Yuri Vyatkin, Ani Azatyan, Georges St. Laurent, Philipp Kapranov, Peter G. Zaphiropoulos

**Affiliations:** ^1^ Department of Biosciences and Nutrition Karolinska Institutet Huddinge Sweden; ^2^ St. Laurent Institute Cambridge MA USA; ^3^ AcademGene LLC Novosibirsk Russia; ^4^ Institute of Genomics Huaqiao University Xiamen China

**Keywords:** CRISPR/Cas9, GLI1/FOXS1 interplay, Hedgehog signaling, medulloblastoma, rhabdomyosarcoma

## Abstract

Hedgehog (HH) signaling is involved in many physiological processes, and pathway deregulation can result in a wide range of malignancies. Glioma‐associated oncogene 1 (GLI1) is a transcription factor and a terminal effector of the HH cascade. Despite its crucial role in tumorigenesis, our understanding of the GLI1 cellular targets is quite limited. In this study, we identified multiple new GLI1 target genes using a combination of different genomic surveys and then subjected them to in‐depth validation in human cancer cell lines. We were able to validate >90% of the new targets, which were enriched in functions involved in neurogenesis and regulation of transcription, in at least one type of follow‐up experiment. Strikingly, we found that RNA editing of GLI1 can modulate effects on the targets. Furthermore, one of the top targets, *FOXS1*, a gene encoding a transcription factor previously implicated in nervous system development, was shown to act in a negative feedback loop limiting the cellular effects of GLI1 in medulloblastoma and rhabdomyosarcoma cells. Moreover, *FOXS1* is both highly expressed and positively correlated with *GLI1* in medulloblastoma samples of the Sonic HH subgroup, further arguing for the existence of FOXS1/GLI1 interplay in human tumors. Consistently, high *FOXS1* expression predicts longer relapse‐free survival in breast cancer. Overall, our findings open multiple new avenues in HH signaling pathway research and have potential for translational implications.

**Abbreviations**
DAPI: 4′,6‐diamidino‐2‐phenylindole
DNMT3B: DNA methyltransferase 3 beta
EdU: 5‐ethynyl‐2′‐deoxyuridine
ENC1: ectodermal‐neural cortex 1
FOXS1: forkhead box S1
GLI1: glioma‐associated oncogene 1
GO: gene ontology
HEPM: human embryonic palatal mesenchyme
HH: Hedgehog
HHIP: human Hedgehog‐interacting protein
PLAT: plasminogen activator tissue type
PTCH: Patched
RNA‐seq: RNA sequencing
RPKM: Reads per kilobase million
SAG: SMO agonist
SHH: Sonic Hedgehog
SMO: Smoothened
SMS: single molecule sequencing
SOSTDC1: Sclerostin Domain Containing 1

## 1 Introduction

The Hedgehog (HH) signaling pathway is a highly conserved signal transduction cascade, implicated in embryonic development, stem cell maintenance, cell cycle progression, apoptosis, and epithelial‐to‐mesenchymal transition (Teglund and Toftgård, 2010). Deregulation of HH signaling is linked to a wide range of malignancies, including basal cell carcinoma, medulloblastoma, rhabdomyosarcoma, glioma, gastrointestinal, pancreatic, prostatic, small‐cell lung, and breast cancer (Briscoe and Thérond, 2013; O'Toole *et al*., 2009; Robbins *et al*., 2012; Teglund and Toftgård, 2010; Tostar *et al*., 2010).

Glioma‐associated oncogene 1 (GLI1) is one of the three GLI transcription factors within the HH pathway (Briscoe and Thérond, 2013; Cohen, 2010; Robbins *et al*., 2012; Teglund and Toftgård, 2010). In addition to being a terminal effector, GLI1 acts also as a GLI target gene, eliciting signal amplification (Teglund and Toftgård, 2010; Varjosalo and Taipale, 2008). Moreover, the expression of GLI1 and its activity as a transcription factor are regulated by both transcriptional and post‐transcriptional processes. Prominent among these are alternative splicing (Lo *et al*., 2009; Shimokawa *et al*., 2008; Zhu *et al*., 2014), interactions with microRNA and antisense RNA (Ferretti *et al*., 2008; Lee *et al*., 2007; Villegas *et al*., 2014), and RNA editing (Shimokawa *et al*., 2013). These mechanisms increase the functional diversity of the GLI1 protein, modulating GLI1‐dependent biological outcomes.

In our previous study (Shimokawa *et al*., 2013), it was shown that the GLI1 mRNA can be RNA edited via adenosine deamination at nucleotide 2179 converting it to inosine and leading to an arginine to glycine change at amino acid 701. Compared to GLI1, edited GLI1 (GLI1‐701G) exhibits a slightly higher transcriptional activation and less sensitivity toward inhibition by the negative regulator of the HH pathway, SUFU (Shimokawa *et al*., 2013). However, GLI1‐701G is less effective in promoting cell growth in medulloblastoma cells. Consistently, about 50% of GLI1 transcripts are edited in human normal tissues, including cerebellum, skin, pancreas, ovary, and colon; on the other hand, in the corresponding tumors and tumor cell lines, the extent of GLI1 editing is reduced (Shimokawa *et al*., 2013). These findings might suggest that RNA editing of GLI1 can have a negative impact on GLI1‐dependent tumorigenesis. However, it was recently shown that GLI1 editing is associated with multiple myeloma development, highlighting the context‐specific impact of this GLI1 post‐transcriptional modification in tumor biology and suggesting that additional investigation into this complex phenomenon is required (Lazzari *et al*., 2017).

Despite the prominence of HH signaling in early development and malignancy, the number of confirmed targets of the GLI1 transcription factor, such as Patched (*PTCH1* and *PTCH2*) and Human Hedgehog‐Interacting Protein (*HHIP*), is surprisingly small (Katoh and Katoh, 2009). In this study, we identified and validated up to 29 novel targets of GLI1 genome‐wide in a rhabdomyosarcoma cell line. Furthermore, we show the effect of RNA editing of GLI1 on the regulation of some of these targets. Strikingly, one of the highly up‐regulated targets, *FOXS1*, was found to be involved in feedback mechanisms that constrain the capacity of GLI1 to act as a proliferation factor, pinpointing to a tight regulatory control of HH signaling. The forkhead family transcription factor FOXS1 is known to be expressed in the sensory nervous system, but absent in other placode and neural crest‐derived cell types (Montelius *et al*., 2007). Importantly, we now demonstrate a prominent *FOXS1* expression in Sonic HH medulloblastomas. Our results shed new light on the importance of *FOXS1* in HH signaling regulation and malignant transformation.

## 2 Materials and methods

### 2.1 Cell lines and culture

The embryonal rhabdomyosarcoma Rh36 cell line was a kind gift from P. Houghton (St. Jude Children's Research Hospital, Memphis, TN, USA). The medulloblastoma Daoy cell line was a kind gift from F. Aberger (University of Salzburg, Austria). Rh36 cells were cultured in RPMI‐1640 Medium + 10% FBS, Daoy cells in EMEM + 10% FBS, and HEK293A (human embryo kidney) cells in Dulbecco's modified Eagle's medium + 10% FBS. Daoy cells were treated with 200 nm SAG in 0.5% FBS and harvested after 48 h. The human embryonal palatal mesenchyme (HEPM) cell line was purchased from ATCC (Manassas, VA, USA) and cultured in EMEM Medium + 10% FBS. All cell lines were maintained in a 5% CO_2_ humidified incubator.

### 2.2 Transfection of cell lines

Predesigned GLI1 siRNA (Villegas *et al*., 2014) and nontargeting control siRNA (SIC001) were purchased from Sigma‐Aldrich (St. Louis, MO, USA), while siRNA targeting FOXS1 (sc‐75023) were purchased from Santa Cruz Biotechnologies (Dallas, TX, USA). Cells were plated in 6‐ or 24‐well plates at 50–70% confluency, and transfections were performed with Lipofectamine RNAimax (Invitrogen, Carlsbad, CA, USA) following the protocol provided by the manufacturer. Rh36 cells were transfected with expression constructs for GLI1 (Shimokawa *et al*., 2013), GLI1‐701G (Shimokawa *et al*., 2013), and 8 control pCMV‐based constructs or siRNA that did not cause significant change in GLI1 mRNA level. Transfections were performed using Lipofectamine LTX (Invitrogen) following manufacturer's instructions.

### 2.3 RNA isolation, cDNA synthesis, and real‐time qPCR

Total RNA from cells was prepared with EZNA^®^ Total RNA Kit I (R6834‐02; Omega Bio‐tek, Norcross, GA, USA) followed by cDNA synthesis with random N6 primers (New England Biolabs, Ipswich, MA, USA) and SuperScript III (Invitrogen). RT‐qPCR was carried out with the FastStart Universal SYBR Green Master (Rox) (Roche, Basel, Switzerland) on a 7500 fast real‐time PCR system (Applied Biosystems, Foster City, CA, USA). For *LOC100507346* (*PTCH1* antisense transcript), to avoid co‐amplification of *PTCH1*, the primers were designed to map to *PTCH1* intron 15, the forward primer positioned at exon 1, and the reverse primer at the junction of exon 1 and exon 2 of *LOC100507346* (Fig. S1A). The other primers were designed using the NCBI primer blast tool (Table S1). All amplifications were run at least in triplicate, and the fold change was normalized to the average expression of the housekeeping genes, *TBP* and *RPLPO*. The relative expression was determined by the $2^{-\Delta \Delta C_{t}}$ method. For the adenoviral transduction experiments, the RNA expression is shown as log$2^{-\Delta \Delta C_{t}}$ in order to minimize the variability of biological replicates. Data were analyzed with graphpad prism 6 (La Jolla, CA, USA) using the Student's *t*‐test.

### 2.4 Helicos single molecule sequencing (SMS)

Sample preparation: RNA isolated from Rh36 cells were first treated with DNase I (Roche) and then subjected to rRNA depletion using RiboZero rRNA Removal kit (Epicentre, Madison, WI, USA). The rRNA‐depleted RNA samples were prepared for the SMS essentially as described previously (Kapranov *et al*., 2010). Sequencing was performed at the SeqLL, LLC facility (Woburn, MA, USA).

#### 2.4.1 Data analysis

Single molecule sequencing reads were processed essentially as described before (Giladi *et al*., 2010) and aligned to the HG19 version of the human genome using indexDPgenomic aligner (Giladi *et al*., 2010). Uniquely aligned reads were used to generate reads per kilobase million (RPKM) for each transcript annotated in the UCSC Genes database (Kent *et al*., 2002). To determine transcripts up‐regulated in the GLI1 or GLI1‐701G over‐expression experiment, we calculated *Z*‐score (*Z*_*j*_) for each transcript *j*:

$$Z_{j}=\frac{{\text{GLI}}_{j}-{\text{CTL}}_{j}}{{\text{SDTL}}_{j}},$$

where: GLI_*j*_: RPKM of the transcript *j* in either GLI1 or GLI1‐701G sample, CTL_*j*_: mean count of the transcript *j* in all eight control samples, SDTL_*j*_: standard deviation of the RPKM of the transcript *j* in the control samples.

To identify transcripts down‐regulated in the two GLI1 depletion experiments, we calculated the ratio of the RPKM counts in each of the GLI1 siRNA vs. the respective control siRNA treatment. Gene ontology (GO) enrichment analysis was based on GOstat package in r environment (http://www.R-project.org) (RC, 2014).

### 2.5 Construction of adenovirus expressing GLI1/GLI1‐701G vectors

The pAd‐Easy system was used for generating recombinant adenoviruses (Luo *et al*., 2007). pAdTrack‐CMV was a gift from Bert Vogelstein (Addgene, Cambridge, MA, USA; plasmid #16405) (He *et al*., 1998), and pCMV‐GLI1‐flag/pCMV‐GLI1‐701G‐flag were constructed in our laboratory (Shimokawa *et al*., 2013). Briefly, pCMV‐GLI1/GLI1‐701G‐flag was double digested with *Bgl*II/*Sal*I and cloned into pAdTrack‐CMV to generate pAdTrack‐CMV‐GLI1/GLI1‐701G. After digestion with *Pac*I, pAdTrack‐CMV‐GLI1/GLI1‐701G‐flag was used to transform electroporation competent BJ5183‐AD‐1 cells and homologously recombined to pAd‐CMV‐GLI1/GLI1‐701G‐flag, which was further transfected into HEK293A cells using Lipofectamine 3000 reagent (Invitrogen) to package adenovirus expressing GLI1/GLI1‐701G‐flag (Ad‐GLI1/GLI1‐701G). Control adenovirus (Ad‐Vector) was constructed using the same method.

### 2.6 Western blot

Cells were plated in 6‐well plate at 50–70% confluency. HEK293A cells were transfected with 2 μg plasmids of pAdTrack‐CMV, pAdTrack‐CMV‐GLI1‐flag, or pAdTrack‐CMV‐GLI1701G‐flag using Lipofectamine 3000 (Invitrogen). Rh36 and Daoy cells were transduced with adenoviruses expressing GLI1, GLI1‐701G, or control adenoviruses. After 48‐h incubation, cells were lysed with RIPA buffer supplemented with Complete Protease Inhibitor Tablets (Roche). Proteins were separated on a 7.5% sodium dodecyl sulfate polyacrylamide gel electrophoresis (SDS/PAGE) followed by transfer to an Immobilon‐P membrane (Millipore, Burlington, MA, USA). The membrane was incubated at 4 °C overnight in StartingBlockTM T20 (TBS) Blocking Buffer (#37543; Thermo Scientific, Waltham, MA, USA) with rabbit anti‐GLI1 antibody (#2553; Cell Signaling Technology, Danvers, MA, USA), rabbit anti‐flag antibody (F7325; Sigma‐Aldrich), rabbit anti‐PTCH1 antibody (GTX108015; GeneTex, Irvine, CA, USA), rabbit anti‐FOXS1 antibody (PA5‐49702; Invitrogen), or mouse anti‐β‐actin (A5441; Sigma‐Aldrich) followed by incubation with goat anti‐rabbit or anti‐mouse secondary antibodies and visualized using Pierce ECL chemiluminescent substrate (Thermo Scientific).

### 2.7 CRISPR/Cas9‐mediated *GLI1* knockout

CRISPR/Cas9‐mediated *GLI1* knockout in Daoy cells was carried out following the protocol of Ran *et al*. (2013), and pSpCas9(BB)‐2A‐GFP(PX458) plasmid was a gift from Feng Zhang (Addgene plasmid #48138). Three different short guide RNAs (sgRNAs) against GLI1 (Table S2) were designed and cloned into the pSpCas9(BB)‐2A‐GFP plasmid, and then, the sequence‐verified CRISPR plasmids pSpCas9(sgRNA)‐2A‐GFP were transfected into 70–80% confluent Daoy cells using Fugene HD (Promega, Madison, WI, USA). After 48‐h incubation, transfected Daoy cells were suspended in EMEM medium + 10% FBS + 1% penicillin/streptomycin, filtered with a cell strainer (BD Biosciences, Stockholm, Sweden), and then sorted in 96‐well plates based on the expression of GFP using a BD FACSAria Fusion cytometer installed with bd facsdiva software (Becton Dickinson, Franklin Lakes, NJ, USA). Single cells were identified by sequential gating, while dead cells were identified based on DAPI (4′,6‐diamidino‐2‐phenylindole) staining.

### 2.8 Cell proliferation

About 30–50% confluent cells per well were seeded in 6‐well plates, treated with siRNA for 48 h, and followed by 2 h (Daoy cells) or 4 h (Rh36 cells) of 10 μm EdU (5‐ethynyl‐2′‐deoxyuridine) incubation. Adenoviruses were added after 6 h of siRNA transfection. EdU was detected by fluorescent‐azide coupling reaction (Click‐iT; Invitrogen), with Alexa fluor 488 azide or Alexa fluor 647 azide (A10277; Life Technologies, Carlsbad, CA, USA) used following siRNA transfection or adenovirus transduction, respectively. For each treatment, 10 000 cells were analyzed on a FACS calibur machine (BD Biosciences). Cell cycle distribution was calculated using the cellquest software (BD Bioscience).

### 2.9 Luciferase reporter assay

HEK293A cells were seeded in 24‐well plates and transfected with 200 ng of 12xGLI binding site luciferase reporter plasmid (12xGLIBS) (Mao *et al*., 2002) and 10 ng of Renilla control reporter plasmid, together with pcDNA3.1 vector, pGL3 basic luciferase empty vector, the pCMV‐GLI1‐flag expression construct (pGLI1), and/or pcDNA3.1‐FOXS1 (OHu29375; GeneScript, Piscatway, NJ, USA) using Lipofectamine 3000 (Invitrogen). The total amount of plasmid DNA in each well, 610 ng, was adjusted with the addition of the pcDNA3.1 vector. After 48‐h plasmid transfection, luciferase activity was measured using the Dual‐Luciferase Reporter Assay (Promega). Additionally, a luciferase assay was performed with the use of the mouse Gli1 promoter construct (Shimokawa *et al*., 2013) instead of the 12xGLIBS plasmid. Data were analyzed with GraphPad Prism 6 using the Student's *t*‐test.

### 2.10 Immunoprecipitation

HEK293A cells were co‐transfected with pAdTrack‐CMV‐FOXS1‐HA expression plasmid, a construct generated from pAdTrack‐CMV and pcDNA3.1‐FOXS1, and pCMV‐GLI1‐flag. Cells were lysed after 48‐h transfection, and the lysate was incubated with rabbit anti‐flag antibody coupled to Dynabeads^®^ Protein A beads (Life Technologies) for 3 h, at 4 °C. Beads were washed with lysis buffer five times and eluted at 98 °C for 10 min. The eluted sample was loaded in an acrylamide gel by following the western blot protocol and blotted with rabbit anti‐flag or mouse anti‐HA antibody (901513; BioLegend, San Diego, CA, USA).

### 2.11 Chromatin immunoprecipitation

Daoy cells were seeded in 150‐mm dishes and were transduced with adenoviruses expressing GLI1, GLI1‐701G, or control adenoviruses for 48 h. ChIP assays were performed essentially as described (Mohammed *et al*., 2016). Briefly, 5 μg of anti‐flag antibody was conjugated to Dynabeads^®^ Protein A beads, and then, antibody‐bound beads were incubated with sonicated cell lysates. Immunoprecipitated DNA was purified using the QIAquick PCR Purification Kit (Hilden, Germany) and quantified by qPCR. Input DNA was used to produce standard curves, and the ChIP data were converted to percentages of total input. GLI1 binding sites on a 10‐kb promoter region of the *PPAP2B* or *PRDM16* gene were predicted using ConSite (Sandelin *et al*., 2004), a web‐based tool for binding sites prediction, incorporating the Position Specific Frequency Matrices of the GLI1 binding sites (Hallikas *et al*., 2006). The two highest scored binding sites of each gene for which reliable qPCR primers could be designed were selected. The ChIP‐qPCR primer sequences are given in Table S6.

### 2.12 Analysis of human medulloblastoma data

Expression data on human medulloblastoma tumors were analyzed through the St. Jude–Washington University Pediatric Cancer (PeCan) Data Portal (https://pecan.stjude.cloud/home).

### 2.13 Patient survival analysis

The Kaplan–Meier plotting tool (http://kmplot.com/analysis/) was used to evaluate the relapse‐free survival of breast cancer patients expressing low or high levels of FOXS1 (Lanczky *et al*., 2016).

## 3 Results

### 3.1 Identification of GLI1 target genes

**Figure 1:**
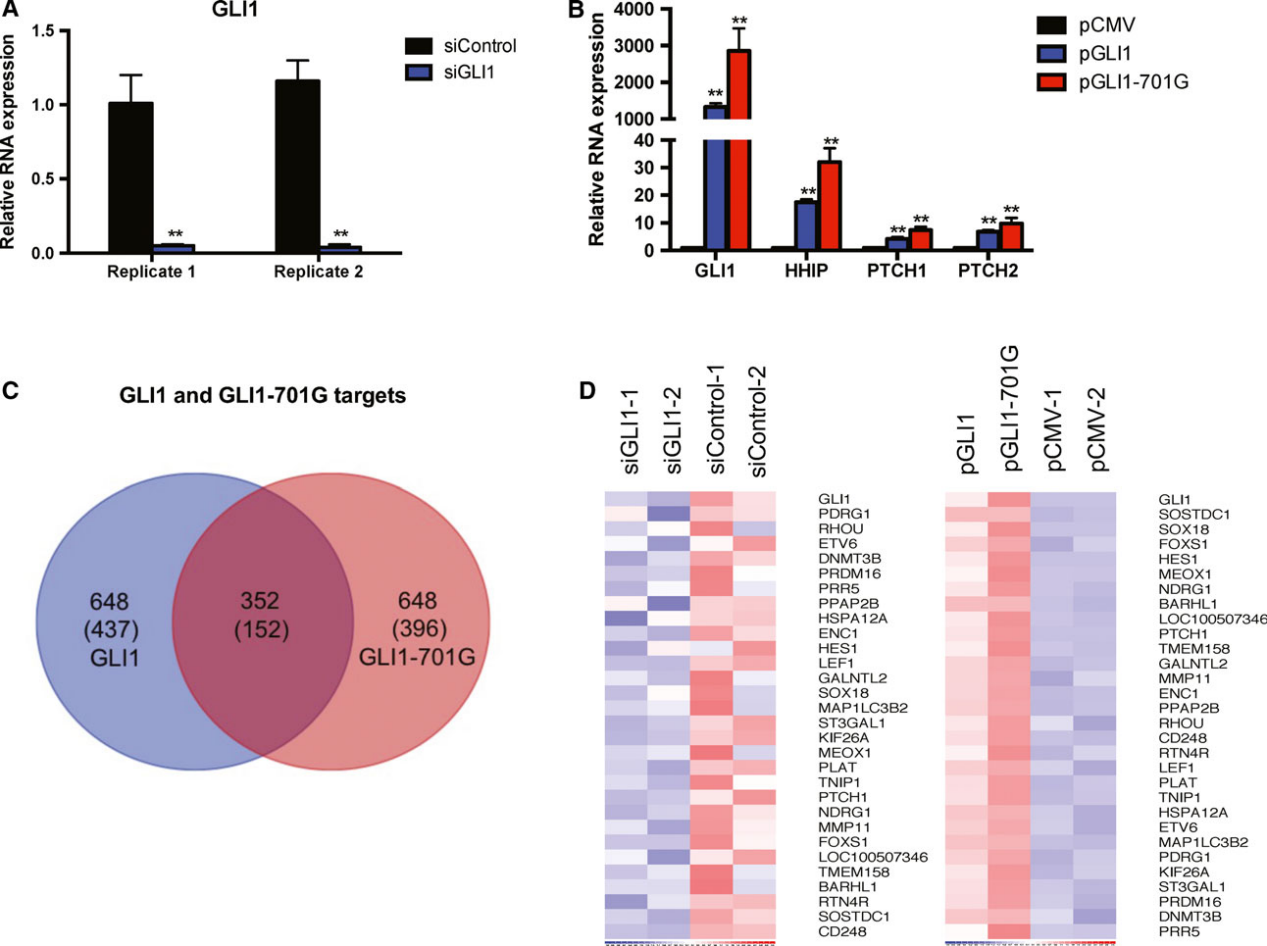
RNA‐seq data analysis. (A) Depletion of *GLI1* in biological replicates of Rh36 cells transfected with siRNA targeting GLI1 (siGLI1) or control siRNA (siControl). (B) Over‐expression of *GLI1, HHIP, PTCH1, PTCH2* in Rh36 cells transfected with pCMV, pGLI1, and pGLI1‐701G. Data from one representative experiment are shown. In panels A and B: *Y*‐axes represent relative expression ($2^{-\Delta \Delta C_{t}}$ values); error bars indicate standard deviation; **P‐value < 0.01, compared to control as calculated by the Student's *t*‐test. (C) Venn diagram representing the 352 common and the 648 unique UCSC transcripts within the datasets of the 1000 highest up‐regulated transcript in GLI1 or GLI1‐701G over‐expression experiments. Numbers in parenthesis refer to the genes corresponding to the transcripts. (D) Heat map analysis of GLI1 and the 29 selected target genes (total 30 genes) in both the knockdown (left) and the over‐expression (right) datasets. The four lanes on the left panel indicate: biological replicates with GLI1 siRNA (siGLI1‐1 and siGLI1‐2) and biological replicates with control siRNA (siControl‐1 and siControl‐2). The four lanes on the right panel indicate: Rh36 cells over‐expressing GLI1 (pGLI1), GLI1‐701G (pGLI1‐701G), biological replicates of empty vector (pCMV‐1 and pCMV‐2). Red and blue colors indicate up‐ and down‐regulation, respectively, with the intensity highlighting the level of up‐ and down‐regulation. Note the clear up‐ and down‐regulation following GLI1 over‐expression/depletion, respectively, compared with 29 randomly selected genes (Fig. S2).

Rhabdomyosarcoma Rh36 cells were transfected with either siRNA targeting GLI1 or plasmids designed to over‐express GLI1 or RNA‐edited version of GLI1 (GLI1‐701G). GLI1 depletion and GLI1/GLI1‐701G over‐expression as well as up‐regulation of the known GLI1 target genes *HHIP*,* PTCH1,* and *PTCH2* in these samples were confirmed by RT‐qPCR (Fig. 1A,B). These samples were then subjected to RNA sequencing (RNA‐seq) analysis using the Helicos SMS platform. In addition, RNA from multiple transfection controls that did not significantly alter GLI1 mRNA level were also subjected to RNA‐seq to ensure that the observed effects were not due to random fluctuations caused by the transfection procedure. Putative target genes up‐regulated in response to GLI1 or GLI1‐701G over‐expression relative to the eight control samples were detected by calculating the respective *Z*‐score (see Section 2). As expected, each of the three GLI1 targets mentioned above was significantly up‐regulated compared to the controls with *Z*‐scores of 34.5 and 37.4 (*HHIP*), 24.8 and 41.6 (*PTCH1*), and 2.5 and 3.4 (*PTCH2*) for GLI1 and GLI1‐701G over‐expression, respectively.

We then selected potential targets of GLI1 using the following steps. First, we ranked UCSC transcripts based on the *Z*‐scores in either GLI1 or GLI1‐701G over‐expression experiments and selected the top 1000 transcripts from each group. The corresponding minimum *Z*‐scores were 4.1 and 5.3 for the top 1000 transcripts in the GLI1 and GLI1‐701G over‐expression, respectively. Second, we identified 352 transcripts representing 152 unique genes common to the two sets of 1000 transcripts (Fig. 1C, Table S3). Third, since GLI1 depletion would also be expected to down‐regulate true targets, we used an additional filter of down‐regulation in siGLI1 versus siControl in both independent GLI1 depletion experiments resulting in the final list of 29 genes (Table 1, Fig. 1D, Fig. S2). The list contained one known target – *PTCH1*; however, because of low expression levels of *HHIP* in Rh36 cells, its down‐regulation in response to GLI1 depletion could not be reliably estimated leading to exclusion from the final list. *PTCH2* was already excluded from the 152‐gene list, as its up‐regulation in response to GLI1 over‐expression did not meet the *Z*‐score cutoff.

**Table 1:** GLI1/GLI1‐701G target genes and their validation in Rh36 and Daoy cells

UCSC ID	Gene Name	Correlation with GLI1 in FANTOM5	Rh36: GLI1 depletion	Rh36: GLI1 over‐expression	Rh36: GLI1‐701G over‐expression	Daoy: GLI1 depletion	Daoy: GLI1 over‐expression	Daoy: GLI1‐701G over‐expression	Daoy: SAG treatment	Number of validated contexts
**uc010mrr.3**	**PTCH1**	**0.47**	+	+	+	+	+	+	+	7
**uc002wwt.1**	**FOXS1**	**0.373**	+	+	+	+	+	+	+	7
**uc001ohm.1**	**CD248**	**0.333**	(+)	(+)	(+)	(+)	+	+	+	3
uc003car.4	GALNTL2	0.331	(+)	+	+	(+)	+	+	+	5
**uc003sth.3**	**SOSTDC1**	**0.32**	+	+	+	+	+	+	(+)	6
uc002zxx.3	MMP11	0.295	(+)	(+)	(+)	+	+	+	(+)	3
uc002idz.3	MEOX1	0.284	+	+	+	(x)	(+)	(+)	(+)	3
uc001cyj.2	PPAP2B	0.277	(+)	+	+	(x)	x	x	+	3
uc009vlh.3	PRDM16	0.274	+	(+)	(+)	(x)	x	x	(+)	1
uc001yos.4	KIF26A	0.267	+	+	+	+	+	(+)	(+)	5
uc022bkm.1	LOC100507346	0.255	NA	+	+	+	+	+	+	6
uc001lcu.3	HSPA12A	0.252	(+)	(+)	(+)	x	(x)	(x)	(x)	0
uc003xos.2	PLAT	0.21	+	+	+	+	+	+	(+)	6
uc003kdc.4	ENC1	0.202	+	+	+	+	+	+	+	7
uc003hyt.2	LEF1	0.172	(+)	+	+	(+)	+	(+)	(+)	3
**uc011baf.2**	**TMEM158**	**0.17**	NA	(+)	+	(x)	(+)	+	(+)	2
uc002yhs.3	SOX18	0.155	+	+	+	(+)	+	+	(+)	5
uc001htf.3	RHOU	0.147	(+)	+	+	(+)	(+)	(+)	+	3
**uc004cbp.1**	**BARHL1**	**0.126**	NA	NA	NA	NA	NA	NA	NA	NA
uc003ftq.2	HES1	0.114	NA	(+)	+	+	(+)	(+)	+	3
**uc002zru.3**	**RTN4R**	**0.087**	+	(+)	+	+	+	+	(+)	5
**uc003yuf.1**	**NDRG1**	**0.083**	NA	(+)	(+)	+	x	(x)	(+)	1
uc009zwk.1	MAP1LC3B2	0.077	NA	(+)	(+)	(x)	(+)	(+)	(+)	0
uc010gee.3	DNMT3B	0.055	NA	(+)	+	(+)	(+)	+	(+)	2
uc010gzt.1	PRR5	0	NA	(+)	(+)	x	(+)	(+)	+	1
uc003yum.2	ST3GAL1	−0.052	NA	+	+	+	+	+	+	6
uc002wxd.3	PDRG1	−0.094	NA	NA	NA	NA	NA	NA	NA	NA
uc001qzz.3	ETV6	−0.131	NA	+	+	(x)	(x)	(x)	+	3
uc011dco.2	TNIP1	−0.247	NA	(x)	(x)	(+)	+	(+)	(+)	1

The Spearman correlation of the list with the FANTOM dataset is 0.172. **Bold** indicates genes down‐regulated more than twofold in GLI1 depletion. ‘+’ indicates genes down‐regulated/up‐regulated by GLI1 depletion/over‐expression or up‐regulated by SAG treatment, reaching statistical significance. ‘(+)’ indicates genes down‐regulated/up‐regulated by GLI1 depletion/over‐expression or up‐regulated by SAG treatment, without reaching statistical significance. ‘x’ indicates genes up‐regulated/down‐regulated by GLI1 depletion/over‐expression, reaching statistical significance. ‘(x)’ indicates genes up‐regulated/down‐regulated by GLI1 depletion/over‐expression or down‐regulated by SAG treatment, without reaching statistical significance. ‘NA’ indicates genes that were not analyzed in the corresponding samples.

Initially, we tested the validity of the final list using a co‐expression approach. Real targets of GLI1 would be expected to correlate with the GLI1 mRNA across a wide range of cell types, and the availability of a broad expression dataset across 833 human cell types and conditions generated by the FANTOM5 consortium allowed us to accomplish the testing of this assumption (Forrest *et al*., 2014). We calculated the Spearman correlation between the GLI1 mRNA and each target transcript as previously described (St Laurent *et al*., 2016). For PTCH1, HHIP, and PTCH2, the correlations were 0.47, 0.4, and 0.27, respectively. As expected for true GLI1 targets, these values were higher than the Spearman correlations with GLI1 of 99.9%, 99.2%, and 90.1% of the other transcripts in the UCSC Genes database. The median Spearman correlation of the 29 genes was 0.172 (Table 1) – higher than expected from a random set of 29 genes in the FANTOM5 dataset (*P*‐value < 0.01, permutation analysis) and suggesting that this list is indeed enriched for true GLI1 targets.

Interestingly, the top two terms in the GO analysis of the 29‐gene were ‘neurogenesis’ (*P*‐value 3.24E‐6, represented by 11 genes) and ‘regulation of transcription from RNA polymerase II promoter’ (*P*‐value 4.25E‐6, represented by 12 genes) (Table S4). Indeed, 8/29 genes in the list encoded transcription factors: *FOXS1*,* MEOX1*,* PRDM16*,* LEF1*,* SOX18*,* BARHL1*,* HES1,* and *ETV6*. Another gene *DNMT3B* encodes DNA Methyltransferase 3 Beta involved in *de novo* DNA methylation. Some other genes like *SOSTDC1*,* PPAP2B,* and *RHOU* encoded functions involved in signal transduction. Overall, this list had multiple genes encoding intriguing functions potentially involved in downstream effects of the GLI1 signaling. Thus, we further subjected each target to in‐depth validation as described below.

### 3.2 Validation of the top potential GLI1 targets following siRNA knockdown in rhabdomyosarcoma and medulloblastoma bio‐replicas

**Figure 2:**
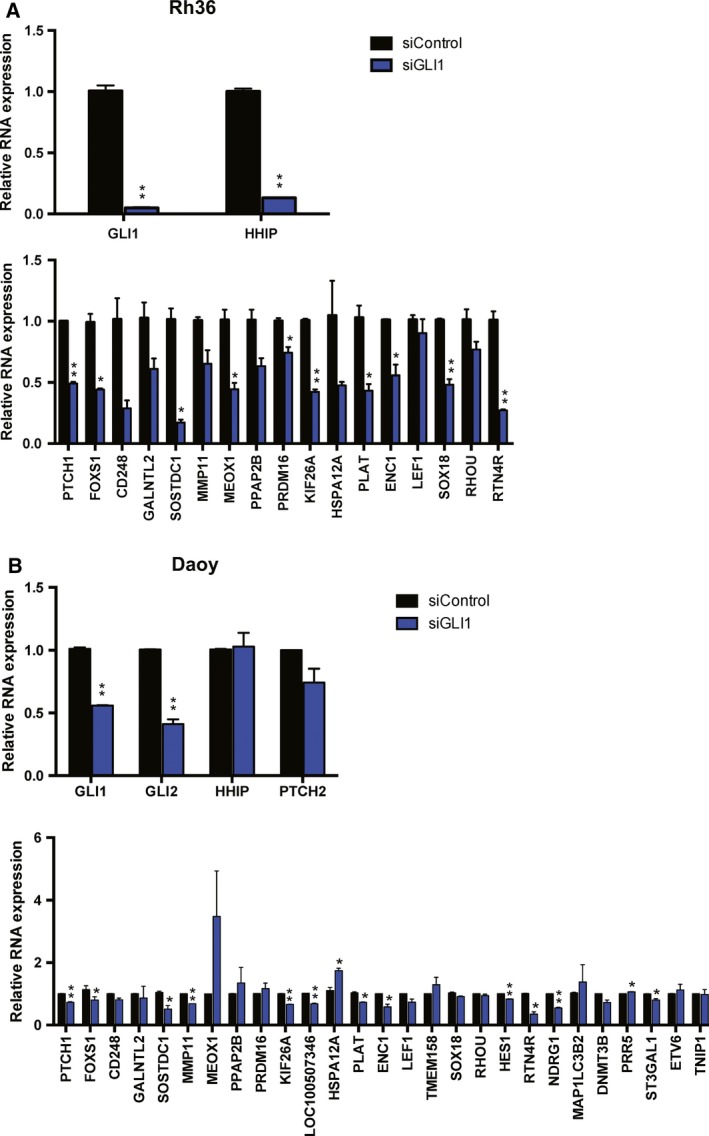
Validation of GLI1 target genes following siRNA knockdown. (A) RNA expression in biological duplicate experiments of *GLI1, HHIP,* and 17 selected targets in Rh36 cells transfected with siRNA targeting GLI1 (siGLI1) or control siRNA (siControl). (B) RNA expression in biological duplicate experiments of *GLI1, GLI2, HHIP, PTCH2*, and 27 GLI1 target genes in Daoy cells transfected with siRNA targeting GLI1 (siGLI1) or control siRNA (siControl). *Y*‐axes represent relative expression ($2^{-\Delta \Delta C_{t}}$ values). Error bars indicate standard error of the mean. Statistical significant, *P < 0.05 and **P < 0.01, compared to control, calculated by the Student's *t*‐test.

Among the 29 selected target genes, RT‐qPCR analysis of *BARLH1* and *PDRG1* expression in Rh36 cells resulted in very high CT values (data not shown) and these were excluded from further analysis. Out of the remaining 27 genes, 17 genes with relatively high correlation with GLI1 in the FANTOM5 dataset (Table 1) were selected and analyzed following GLI1 depletion (Fig. 2A). All genes were down‐regulated by GLI1 depletion, with 10 reaching statistical significance in two independent biological experiments, different from the ones used in RNA‐seq.

To address the impact of GLI1 depletion on the 27 target genes in another cellular context, the medulloblastoma Daoy cell line was used. Consistently, GLI1 silencing elicited a reduction of expression in 12 genes that reached statistical significance in two independent biological experiments (Fig. 2B), with the notable exception of *HSPA12A* and *PRR5*, which were up‐regulated. Interestingly, seven of these genes, *PTCH1*,* FOXS1, SOSTDC1, KIF26A, PLAT, ENC1,* and *RTN4R*, were also found in the Rh36 cell validation analysis (Table 1), highlighting a robustness of the GLI1 target gene list.

### 3.3 Validation of GLI1 targets following over‐expression of GLI1/GLI1‐701G in rhabdomyosarcoma and medulloblastoma bio‐replicas

To address the impact of GLI1 and GLI1‐701G on the selected targets, we shifted from the standard transfection of GLI1/GLI1‐701G expression constructs that were used in RNA‐seq to the adenoviral system, as this can sustain high exogenous gene expression. Western blot analysis demonstrated a comparable protein expression of GLI1 and GLI1‐701G from the adenoviral constructs in HEK293A cells (Fig. S3A), and a comparable GLI1, FOXS1, and PTCH1 expression in Rh36 and Daoy cells transduced with adenovirus expressing GLI1/GLI1‐701G (Fig. S3B,C).

**Figure 3:**
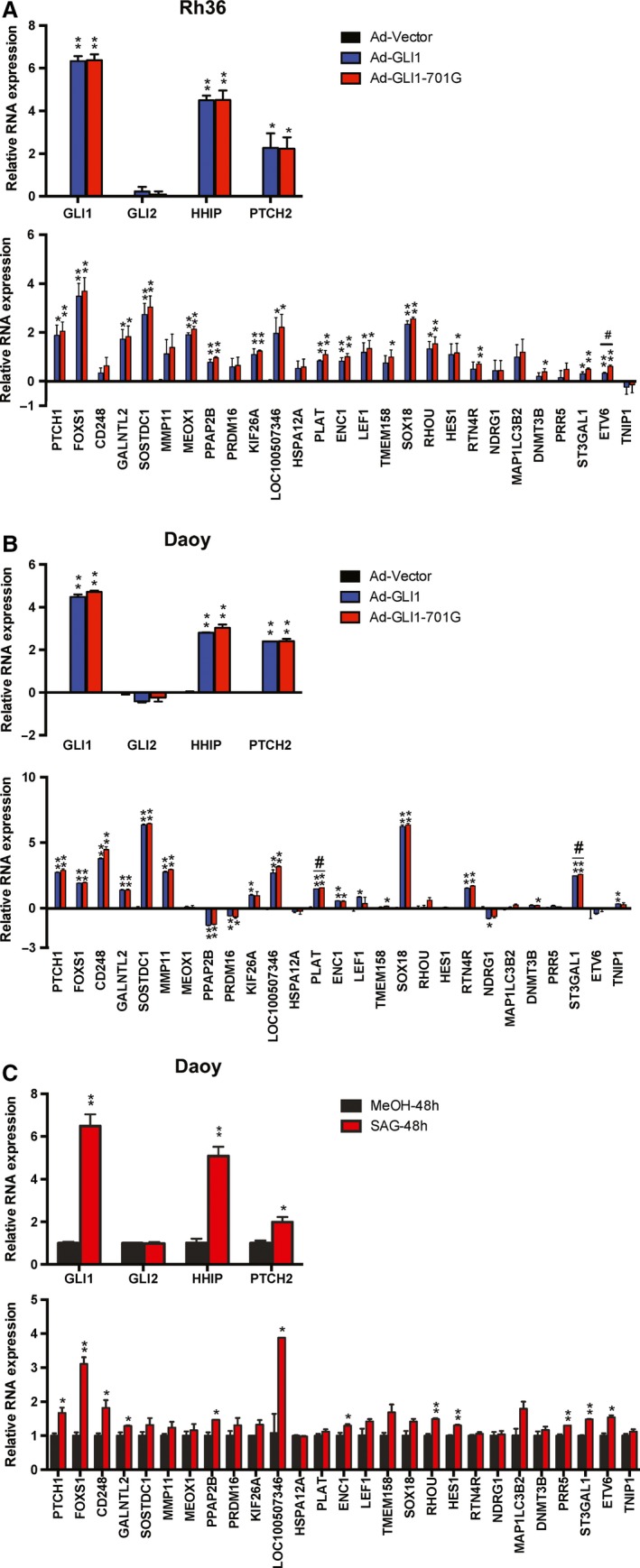
Validation of GLI1/GLI1‐701G target genes following transduction of adenoviruses. RNA expression of *GLI1, GLI2, HHIP, PTCH2,* and 27 GLI1 target genes in Rh36 cells (A) and Daoy cells (B), following transduction with adenoviruses expressing GLI1 (Ad‐GLI1), GLI1‐701G (Ad‐GLI1‐701G), or control adenoviruses (Ad‐Vector). Data from biological triplicate (Rh36) or duplicate (Daoy) experiments are represented as relative expression (log$2^{-\Delta \Delta C_{t}}$ values). Note that the values from the Ad‐Vector group are represented by black bars, but in most cases, these are hardly visible. Error bars indicate standard error of the mean. Statistical significant, *P < 0.05 and **P < 0.01, compared to control, calculated by the Student's *t*‐test. ^#^ *P* < 0.05 indicates statistical significance between Ad‐GLI1 and Ad‐GLI1‐701G groups. (C) RNA expression of *GLI1, GLI2, HHIP, PTCH2,* and 27 GLI1 target genes in Daoy cells treated with the addition of 200 nm SAG for 48 h. Data from one representative experiment are shown as relative expression ($2^{-\Delta \Delta C_{t}}$ values). Error bars indicate standard deviation. Statistical significant, *P < 0.05 and **P < 0.01, compared to control, calculated by the Student's *t*‐test.

Transduction of Rh36 cells revealed statistically significant up‐regulation of 15 out of the 27 targets by both GLI1 and GLI1‐701G in three independent biological experiments, with *FOXS1*,* SOSTDC1,* and *SOX18* being the most responsive genes (Fig. 3A, Table 1). Consistently, standard transfection of increasing amounts of the GLI1 or GLI1‐701G expression constructs in Rh36 cells revealed a dose‐dependent up‐regulation of *FOXS1*,* SOSTDC1,* and *SOX18* (Fig. S3D,E).

In Daoy cells, adenoviral transduction resulted in statistically significant up‐regulation of 12 genes by both GLI1 and GLI1‐701G in two independent biological experiments (Fig. 3B, Table 1), nine of which in common with the same assay on Rh36 cells: *PTCH1*,* FOXS1*,* GALNTL2*,* SOSTDC1*,* LOC100507346*,* PLAT*,* ENC1*,* SOX18,* and *ST3GAL1*. Moreover, 12 genes, *PTCH1*,* FOXS1*,* CD248*,* GALNTL2*,* PPAP2B*,* LOC100507346*,* ENC1*,* RHOU*,* HES1*,* PRR5*,* ETV6*,* ST3GAL1*, were statistically significant up‐regulated in Daoy cells by treatment with the synthetic small molecule Smoothened (SMO) agonist SAG, which activate the HH pathway signaling molecule SMO (Frank‐Kamenetsky *et al*., 2002) (Fig. 3C, Table 1).

The results summarized in Table 1 show that three genes, *PTCH1*,* FOXS1,* and *ENC1*, were successfully validated in all seven tested cellular contexts, following GLI1 depletion or GLI1/GLI1‐701G over‐expression in Rh36 and Daoy cells and SAG treatment in Daoy cells. Two genes, *SOSTDC1* and *PLAT*, were validated in 6 out of the 7 tested contexts. Moreover, *LOC10050734*, a noncoding gene antisense to *PTCH1* (Fig. S1A), and *ST3GAL1* were validated in 6 out of 6 tested contexts. Additionally, four genes, *GALNTL2*,* KIF26A*,* SOX18,* and *RTN4R,* were validated in 5 out of the 7 tested contexts. Therefore, these genes represent the most consistent GLI1 targets.

### 3.4 Differential target genes of GLI1 and GLI1‐701G

Interestingly, some of the identified targets responded differently to the edited versus the nonedited version of GLI1 (Fig. 3A,B). Specifically, in Rh36 cells, *ETV6* was up‐regulated by both GLI1 and GLI1‐701G; however, the latter had higher statistically significant effect (Fig. 3A, Table S5). Additionally, the expression of four genes, *TMEM158*,* HES1*,* RTN4R,* and *DNMT3B*, was significantly up‐regulated by only GLI1‐701G (Fig. 3A, Table 1, Table S5).

Moreover, in Daoy cells, *PLAT* and *ST3GAL1* were up‐regulated by both GLI1 and GLI1‐701G, and however, the latter had higher statistically significant effect (Fig. 3B, Table S5). Additionally, the expression of two genes, *TMEM158* and *DNMT3B*, was significantly up‐regulated by only GLI1‐701G. Importantly, *TMEM158* and *DNMT3B* were also identified in the respective analysis of the Rh36 cells. On the contrary, the expression of three genes, *KIF26A*,* LEF1,* and *TNIP1*, was significantly up‐regulated by only GLI1. Finally, two genes, *PRDM16* and *PPAP2B*, were instead down‐regulated by GLI1 and GLI1‐701G, while *NDRG1* significantly down‐regulated by only GLI1 (Fig. 3B, Table 1, Table S5). ChIP assays revealed that binding of GLI1/GLI1‐701G to the promoter of *PPAP2B* was not detectable, while binding to the *PRDM16* promoter was very weak, compared to the promoter of *PTCH1*, which is the classical GLI1 target gene and served as a positive control (Fig. S3F).

Taken together, the data are supportive of the selected gene list in representing a signature of GLI1 targets and highlight cell context differences, as well as similarities, between GLI1 and GLI1‐701G in their capacity to regulate gene expression.

### 3.5 Reciprocal regulation of *GLI1* and *FOXS1* expression

**Figure 4:**
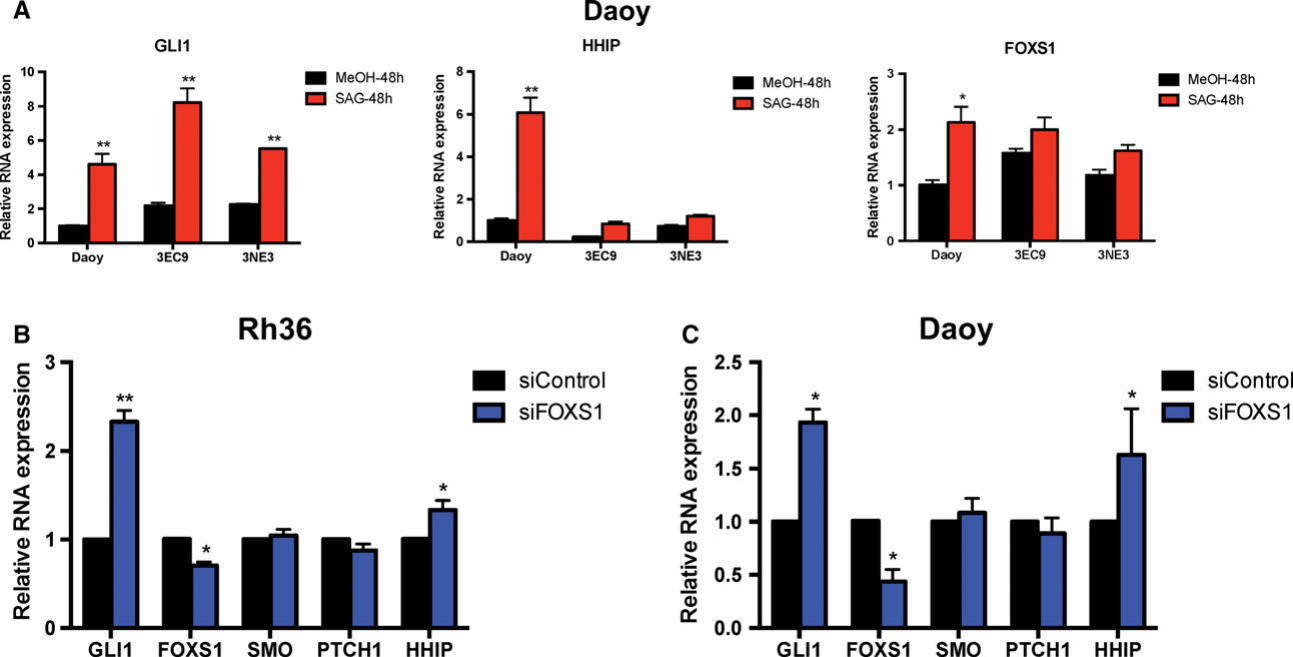
Reciprocal regulation of *GLI1* and *FOXS1* expression. (A) RNA expression of *GLI1*,* HHIP,* and *FOXS1* in Daoy cells and CRISPR/Cas9 mediated *GLI1* knockout Daoy subclones (3EC9 and 3NE3) treated with 200 nm SAG or Methanol (MeOH) for 48 h. Data from one representative experiment are shown as relative expression ($2^{-\Delta \Delta C_{t}}$ values). Error bars indicate standard deviation. Statistical significant, *P < 0.05 and **P < 0.01, compared to control, calculated by the Student's *t*‐test. RNA expression of *GLI1, FOXS1, SMO, PTCH1,* and *HHIP* in Rh36 cells (B) and Daoy cells (C) transfected with siRNA targeting FOXS1 (siFOXS1) or control siRNA (siControl). Data from biological triplicate experiments are shown as relative expression ($2^{-\Delta \Delta C_{t}}$ values). Error bars indicate standard error of the mean. Statistical significant, *P < 0.05 and **P < 0.01, compared to control, calculated by the Student's *t*‐test.

To further corroborate on the finding that *FOXS1* is a target of GLI1, we knocked out *GLI1* in the HH signaling‐responsive Daoy cells using CRISPR/Cas9 technology, generating subclones 3EC9 and 3NE3 (Fig. S4A,B). As expected, treatments with SAG for 48 or 72 h up‐regulated *GLI1*,* HHIP,* and *FOXS1* in Daoy cells (Fig. 4A, Fig. S4C). The same treatment of the two CRISPR/Cas9 Daoy subclones increased expression of the mutant endogenous *GLI1* gene, but no longer up‐regulated *HHIP* and *FOXS1* (Fig. 4A, Fig. S4C). Thus, *FOXS1* up‐regulation by the small molecule activator of the HH pathways requires *GLI1*.

Considering that GLI1 regulates itself and thus represents an example of a feed‐forward amplification loop in the HH signaling cascade (Regl *et al*., 2002), we explored the possibility of additional signaling loops within the pathway and whether the newly identified targets of GLI1 participate in them. We chose two highly responsive genes *FOXS1* and *SOSTDC1* for this analysis and tested the effect of their depletion on GLI1 expression. SOSTDC1 depletion in Rh36 cells had little influence on the expression of HH signaling components (Fig. S4D). However, FOXS1 knockdown resulted in increased *GLI1* and *HHIP* expression, while *SMO* and *PTCH1* expression was unaffected, in both Rh36 (Fig. 4B) and Daoy cells (Fig. 4C). These observations indicate that a FOXS1‐GLI1 interplay may represent an example of a novel negative feedback loop in the HH signaling cascade.

### 3.6 Reciprocal proliferation effects of GLI1 and FOXS1

**Figure 5:**
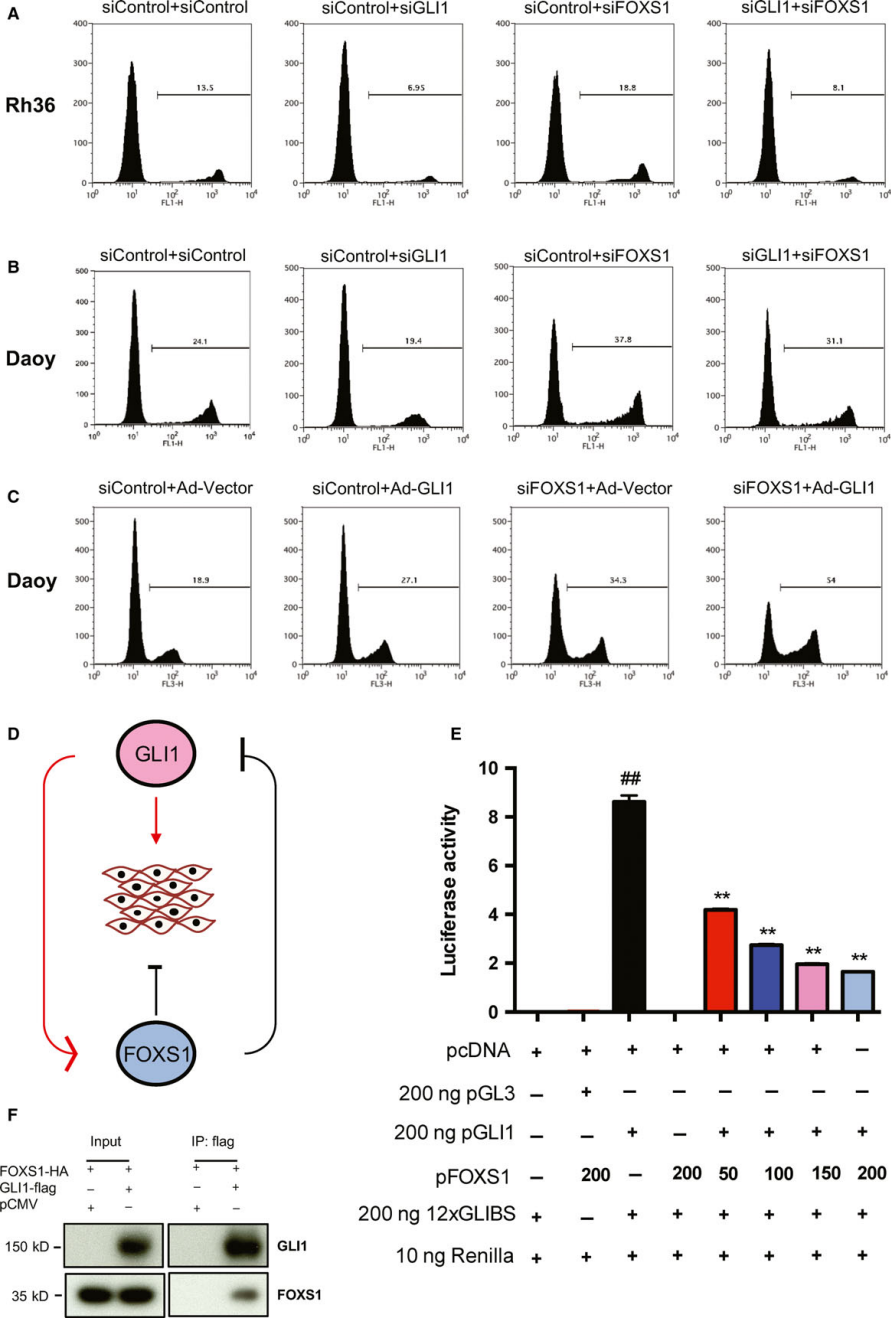
Reciprocal proliferation effects of GLI1 and FOXS1; FOXS1 inhibits GLI1 activity. EdU incorporation assay of Rh36 cells (A) and Daoy cells (B) following transfection with siRNA combinations of siControl + siControl, siControl + siGLI1, siControl + siFOXS1, or siGLI1 + siFOXS1. Data from one representative experiment are shown in the histogram. EdU incorporation assay of Daoy cells (C) following transfection/transduction with siControl + Ad‐Vector, siControl + Ad‐GLI1, siFOXS1 + Ad‐Vector, or siFOXS1 + Ad‐GLI1. Adenoviruses were added 6 h after siRNA transfection. Data from one representative experiment are shown in the histogram. (D) A schematic diagram of the proposed model for the FOXS1/GLI1 feedback loop. The transcription factor GLI1 positively regulates the expression of the FOXS1 transcription factor, while FOXS1 negatively regulates GLI1. GLI1 promotes cell proliferation, whereas FOXS1 inhibits cell proliferation. (E) FOXS1 expression reduces GLI1 transcriptional activity. HEK293A cells were co‐transfected with pcDNA3.1 vector (pcDNA), pGL3 basic luciferase empty vector (pGL3), pCMV‐GLI1‐flag (pGLI1), or pcDNA3.1‐FOXS1 (pFOXS1), together with the reporter plasmid 12xGLIBS and the control plasmid Renilla. Data from one representative experiment are shown. Error bars indicate standard deviation. Statistical significant, ^##^ *P* < 0.01, compared to pcDNA; **P < 0.01, compared to pGLI1, calculated by the Student's *t*‐test. (F) Protein–protein interaction of GLI1 and FOXS1. HEK293A cells were co‐transfected pAdTrack‐CMV‐FOXS1‐HA (FOXS1‐HA) with pCMV vector (pCMV) or pCMV‐GLI1‐flag (GLI1‐flag), and the whole cell extracts were incubated with rabbit anti‐flag antibody. The presence of FOXS1 in the immunoprecipitates was determined using mouse anti‐HA antibody, and GLI1 expression was verified using rabbit anti‐flag antibody.

In line with its impact on GLI1 expression, FOXS1 knockdown increased the proliferation of both Rh36 and Daoy cells, and this contrasts the decreased proliferation elicited by GLI1 knockdown (Fig. 5A,B) (Villegas *et al*., 2014). Similar changes in proliferation were also observed with the human embryonic palatal mesenchyme (HEPM) cell line (Fig. S5A). Double FOXS1/GLI1 knockdown reduced the proliferation increase elicited by FOXS1 knockdown (Fig. 5A,B), suggesting that the *GLI1* expression levels may underlie the proliferation effects mediated by FOXS1 depletion. Additionally, viral transduction of GLI1 in the HH signaling‐responsive Daoy cells, but not in the nonresponsive Rh36 cells, increased cellular proliferation, similarly to FOXS1 depletion (Fig. 5C, Fig. S5B). Importantly, the combined GLI1 transduction/FOXS1 depletion resulted in a further enhancement of proliferation (Fig. 5C). These observations support the notion that GLI1 has a role in the FOXS1 proliferation effects and, moreover, suggest that the increase in *FOXS1* expression elicited by GLI1 can limit the extent of GLI1‐mediated proliferation. Taken together, the data provide evidence for a FOXS1/GLI1 feedback loop, with *FOXS1*, acting as a GLI1 target, constraining the GLI1 cellular effects, via, at least partly, a negative impact on *GLI1* expression (Fig. 5D).

To investigate the potential mechanism of the FOXS1/GLI1 regulation, luciferase assays with a 12xGLI binding site reporter were performed. Although FOXS1 expression alone did not affect luciferase activity, the GLI1‐mediated transcriptional activation was gradually decreased by the increasing amount of FOXS1 expression (Fig. 5E). Additionally, FOXS1 expression reduced the GLI1 transcriptional activation of the mouse Gli1 promoter (Shimokawa *et al*., 2013) (Fig. S5C). These findings indicate that FOXS1 may interact with GLI1 and block GLI1 activity. Protein immunoprecipitation assays confirmed an interaction between FOXS1 and GLI1 (Fig. 5F). The results provide an interpretation to the distinct effects on cell proliferation of FOXS1 and GLI1.

### 3.7 *FOXS1* is highly expressed in the Sonic Hedgehog (SHH) medulloblastoma subgroup

**Figure 6:**
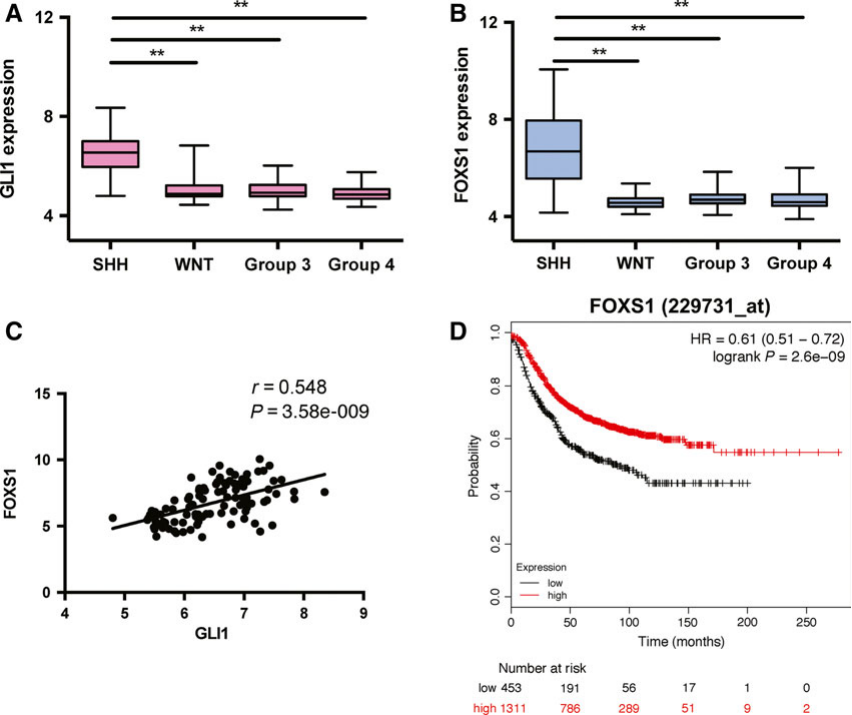
*FOXS1* expression positively correlates with *GLI1* expression in clinical medulloblastoma samples. Box plot analysis of *GLI1* expression (A) and *FOXS1* expression (B) in 392 medulloblastoma tumors, encompassing the four subgroups, SHH (*n* = 100), WNT (*n* = 42), Group 3 (*n* = 101), and Group 4 (*n* = 149), available through the St. Jude PeCan data portal. Data are shown as box plots with horizontal bars representing the maximum, 75th percentile, median, 25th percentile, and minimum values of gene expression. Statistical significant, **P < 0.01, compared to the SHH subtype, calculated by the Mann–Whitney *U*‐test. (C) Scatter plot and Pearson correlation between *GLI1* and *FOXS1* expression in different samples. (D) Relapse‐free survival analysis in breast cancer patients. Kaplan–Meier plot showing that high *FOXS1* expression correlates with better relapse‐free survival (RFS) in patients with breast cancer. The Kaplan–Meier plot is stratified for high (red) and low (black) *FOXS1* expression (*n* = 1764, *P* = 2.6e‐09).

To explore the clinical relevance of FOXS1 on HH signaling‐dependent tumorigenesis, a large medulloblastoma cohort of 392 samples, which includes the four molecular subgroups (SHH signaling, *n* = 100; WNT signaling, *n* = 42; Group 3, *n* = 101; Group 4, *n* = 149) (Taylor *et al*., 2012), was examined (Downing *et al*., 2012). The analysis revealed that the SHH subgroup tumors exhibit high levels of both *GLI1* and *FOXS1* expression, compared to the other three subgroups (Fig. 6A,B). Importantly, the expression of *FOXS1* strongly correlates (*r* = 0.548, *P* = 3.58e‐009) with the expression of *GLI1* (Fig. 6C). Additionally, in the 72 prostate cancer sample dataset (GEO accession: GSE56916), *FOXS1* expression also strongly correlates (*r* = 0.439, *P* < 0.0001) with *GLI1* expression (Fig. S5D). These results further support the notion that *FOXS1* is a target of GLI1, and suggest that an interplay of *GLI1* and *FOXS1* expression is also present in tumors.

## 4 Discussion

In this work, a signature of 29 GLI1 target genes was identified, via a combination of RNA‐seq analyses of GLI1 over‐expression and depletion datasets supplemented with the global co‐expression analysis based on the FANTOM5 dataset. Overall, 25/27 (>90%) tested genes were validated in at least one independent test (Table 1). Five genes, *PTCH1*,* FOXS1*,* SOSTDC1*,* PLAT*, and *ENC1,* were validated in at least 6 out of 7 independent tests, performed on both Rh36 and Daoy cells. PTCH1 is a known GLI1 target and acts as a receptor of HH ligands and a negative regulator of SMO activity. Loss‐of‐function mutations in PTCH1 elicit aberrant activation of the pathway and can lead to basal cell carcinoma (Hahn *et al*., 1996) and medulloblastoma (Pazzaglia *et al*., 2006). FOXS1, Forkhead Box S1, is a transcription factor identified as an early sensory neuronal marker, and its expression is of importance for integration and processing of balance, hearing, and motor functions. Surprisingly, mice lacking Foxs1 expression develop normally and tissues expressing Foxs1 appear normal, without overt phenotypes (Heglind *et al*., 2005; Montelius *et al*., 2007). It has also been reported that *Foxs1* is hypomethylated and up‐regulated in murine postgonadectomy adrenocortical tumors (Schillebeeckx *et al*., 2015). SOSTDC1, Sclerostin Domain Containing 1, is a secreted inhibitor of the WNT and BMP pathways, which plays a role in a WNT‐SHH‐SOSTDC1 negative feedback loop that is involved in the mechanism controlling spatial patterning of teeth in mice, and also acts downstream of SHH signaling (Cho *et al*., 2011). PLAT, Plasminogen Activator Tissue Type, a serine protease, induces the conversion of inert zymogen plasminogen to protease plasmin, which degrades the surrounding matrix, allowing cancer cells to migrate to distant sites (Chandrasekar *et al*., 2003). ENC1, Ectodermal‐Neural Cortex 1, a nuclear matrix protein, is abundantly expressed in the brain (Kim *et al*., 2000) and up‐regulated in human medulloblastoma specimen (Yokota *et al*., 2004). Interestingly, the noncoding *PTCH1* antisense gene *LOC100507346* is also a GLI1 target, which has been validated in 6 out of 6 independent tests. It initiates at *PTCH1* intron 15 on the opposite strand and contains four exons. H3K27ac, a mark associated with active enhancers, is present at the proximal region of the *LOC100507346* transcription start site (UCSC genome browser, https://genome.ucsc.edu) (Fig. S1B). As both PTCH1 and LOC100507346 are GLI1 targets, it is interesting to consider a possible functional interplay of this pair of sense–antisense transcripts and explore potential consequences on HH signaling activity.

On the other hand, additional GLI1 targets almost certainly exist, as the 29 genes were initially identified in a single rhabdomyosarcoma cell line. Consequently, some target genes, including the known GLI1 targets *PTCH2* and *HHIP,* can be missed, as their context‐specific expression may not pass the set thresholds. In this respect, it is worth noting that additional targets of GLI1 might exist in the dataset of 152 genes common to GLI1 and GLI1‐701G, as illustrated by HHIP, which is included in that list, but not in the final 29 genes. However, despite these limitations, this study significantly expands our knowledge on the downstream effectors of the HH signaling pathway.

Concerning the possibility that RNA editing may modulate GLI1 function, it is interesting to note that in both Rh36 and Daoy cells, genes that were preferentially or exclusively regulated by GLI1 or GLI1‐701G were observed. Two of these genes, *TMEM158* and *DNMT3B*, are common in the two cells lines, while the remaining three and six unique to Rh36 and Daoy, respectively, highlighting context‐specific effects of GLI1 editing.

Surprisingly, even though *FOXS1* is a prominent GLI1 target gene, it apparently counteracts the GLI1 cellular effects. *GLI1* is a known oncogene (Nilsson *et al*., 2000) and promotes cellular proliferation. On the other hand, FOXS1 depletion also promotes cellular proliferation, arguing that the increased expression of *FOXS1*, elicited by GLI1 up‐regulation, acts in a negative feedback constraining GLI1 activity. Luciferase reporter and immunoprecipitation assays suggest a potential mechanism on the interplay between FOXS1 and GLI1, as FOXS1 is found to interact with GLI1 and block GLI1 activity. Consequently, *FOXS1* may have tumor suppressive properties and its up‐regulation in tumors could be a marker of good prognosis. In fact, it is possible that a high FOXS1 to GLI1 ratio rather than just high FOXS1 levels better predicts a positive outcome in GLI1‐dependent tumors.

An interesting question in this context is whether the *FOXS1* co‐expression to the *GLI1* oncogene in medulloblastoma and prostate cancer promotes or inhibits tumorigenesis. The data from the analysis of the Rh36 and Daoy cancer cell lines are in line with an inhibitory role. In this direction is also the protective role of *FOXS1* expression in relapse‐free survival of breast cancer (Fig. 6D), another tumor where GLI1 signaling has been implicated (Diao *et al*., 2016). Further work is necessary, though, to conclusively establish such a scenario.

Moreover, additional genes from the 29‐list may also engage in regulatory loops with GLI1 and detailed experimentation is needed to validate this hypothesis. Considering the crucial role of HH signaling in many aspects of human biology, such gene interplays may not be unlikely. In fact, given the current limitations of therapeutic targeting of HH signaling‐dependent tumors and the development of resistance (Rudin *et al*., 2009; Yauch *et al*., 2009) exploiting GLI1 regulatory loops may prove to have substantial benefits.

## 5 Conclusion

This study identified and validated a signature of GLI1 target genes. Additionally, context‐specific differences in the impact of GLI1 and GLI1‐701G on target genes were observed. Moreover, one of the highly up‐regulated targets, *FOXS1*, was found to engage in feedback mechanisms that limit the capacity of GLI1 to act as a proliferation factor. Finally, *FOXS1* expression highly correlated with *GLI1* expression in SHH medulloblastoma. The finding of a FOXS1/GLI1 feedback loop may also provide additional possibilities to develop effective markers for SHH medulloblastoma.

## Author contributions

YD and MF‐UR performed experiments; YD, MF‐UR, AA, and PGZ analyzed experimental results: YV, GSL, and PK analyzed bioinformatic data; YD, MF‐UR, PK, and PGZ wrote the manuscript.

## Supporting information


**Fig. S1.** Genomic organization of *PTCH1* and *LOC100507346*.
**Fig. S2.** Heat map analysis of 29 randomly selected genes.
**Fig. S3.** Validation of GLI1 target genes following over‐expression of GLI1/GLI1‐701G.
**Fig. S4.** The expression of FOXS1 in CRISPR/Cas9 mediated *GLI1* knockout Daoy subclones.
**Fig. S5.** FOXS1 regulates cell proliferation, inhibits GLI1 activity and correlates with GLI1 expression.

**Table S1.** RT‐qPCR primer sequences.

**Table S2.** Sequence of the GLI1 single guide RNAs.

**Table S3.** Top 1000 transcripts upregulated in GLI1/GLI1‐701G over‐expression and the common transcripts in both 1000 lists.

**Table S4.** Enriched GO terms within biological processes using the 29 GLI1 and GLI1‐701G common target genes.

**Table S5.** Differential target genes of GLI1/GLI1‐701G and *P*‐value among different comparisons.

**Table S6.** ChIP‐qPCR primer sequences.
