# Supplementary material for: Identification of novel GLI1 target genes and regulatory circuits in human cancer cells
Source: Mol Oncol. 2018 Aug 30;12(10):1718–34. doi: 10.1002/1878-0261.12366 (PMC6166001; doi:10.1002/1878-0261.12366)
Supplement: Supplementary file 1 — Fig. S1. Genomic organization of PTCH1 and LOC100507346. Fig. S2. Heat map analysis of 29 randomly selected genes. Fig. S3. Validation of GLI1 target genes following over‐expression of GLI1/GLI1‐701G. Fig. S4. The expression of FOXS1 in CRISPR/Cas9 mediated GLI1 knockout Daoy subclones. Fig. S5. FOXS1 regulates cell proliferation, inhibits GLI1 activity and correlates with GLI1 expression. [file MOL2-12-1718-s001.pdf]

Supplementary Fig. S1

A

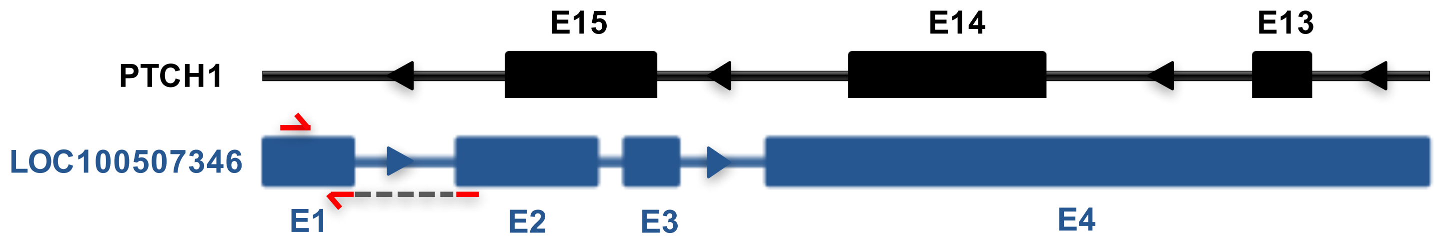

B

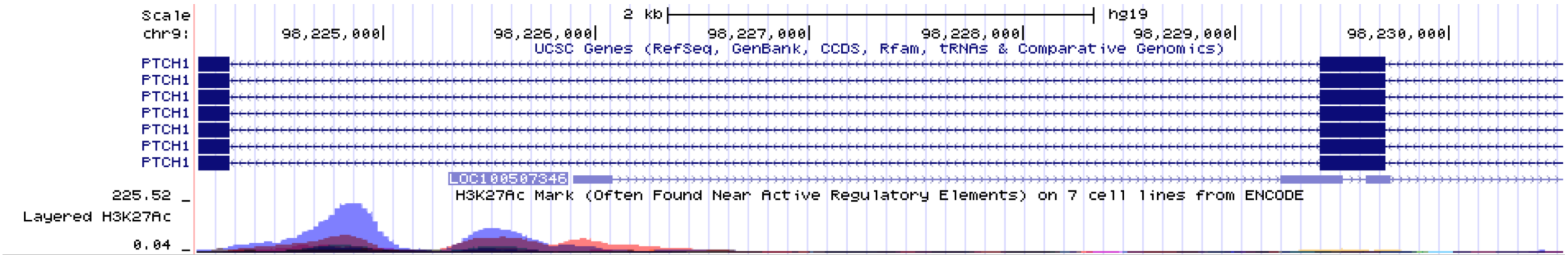

Supplementary Fig. S2

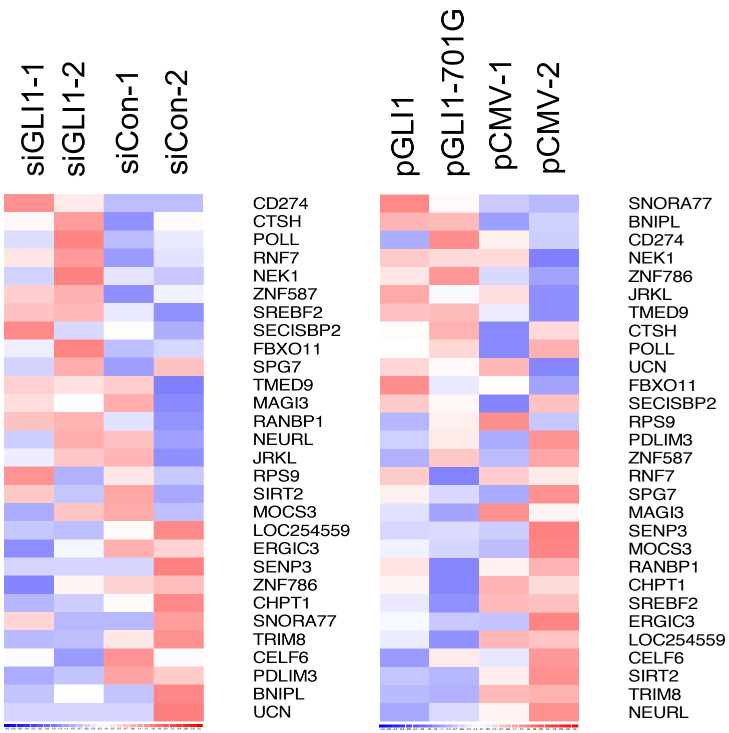

Supplementary Fig. S3

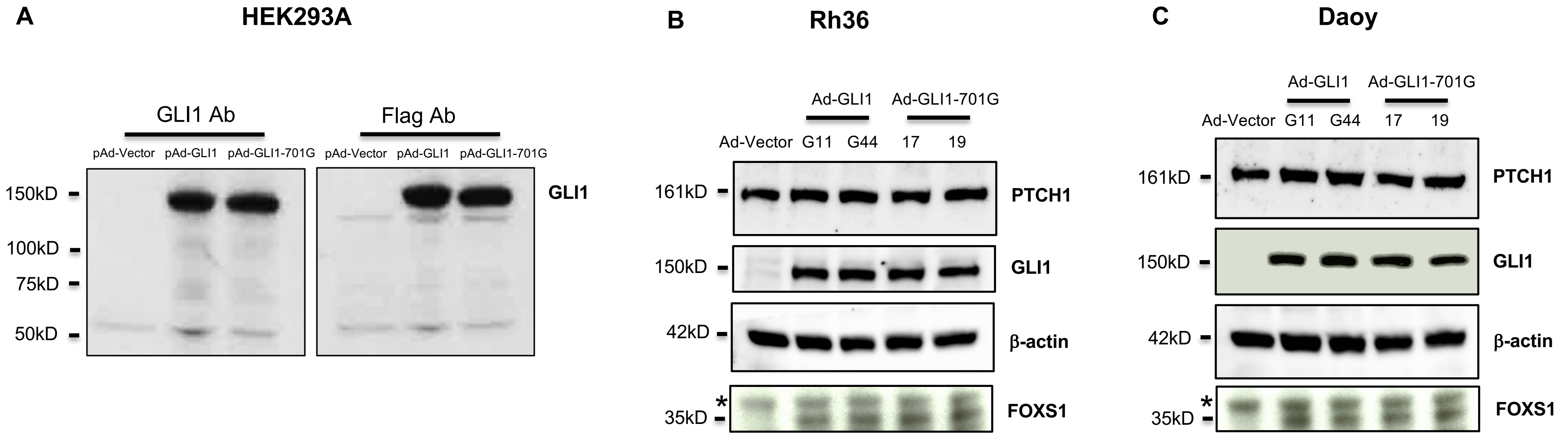

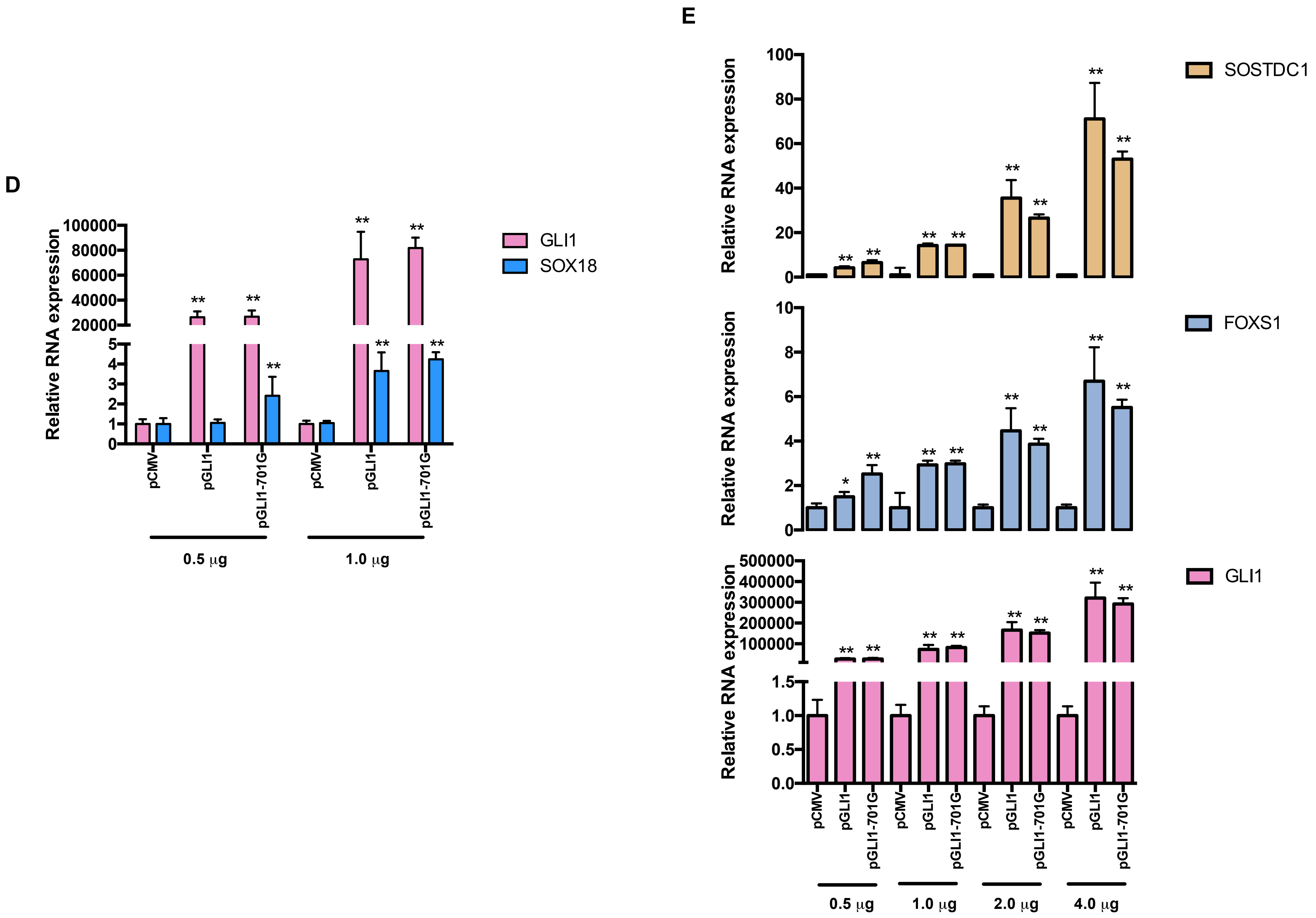

D
Relative RNA expression
100000
80000
60000
40000
20000
5
4
3
2
1
0
GLI1
SOX18
pCMV
pGLI1
pGLI1-701G
0.5 µg
1.0 µg
E
SOSTDC1
FOXS1
GLI1
Relative RNA expression
100
80
60
40
20
10
8
6
4
2
500000
400000
300000
200000
100000
1.5
1.0
0.5
0.0
pCMV
pGLI1
pGLI1-701G
0.5 µg
1.0 µg
2.0 µg
4.0 µg

F

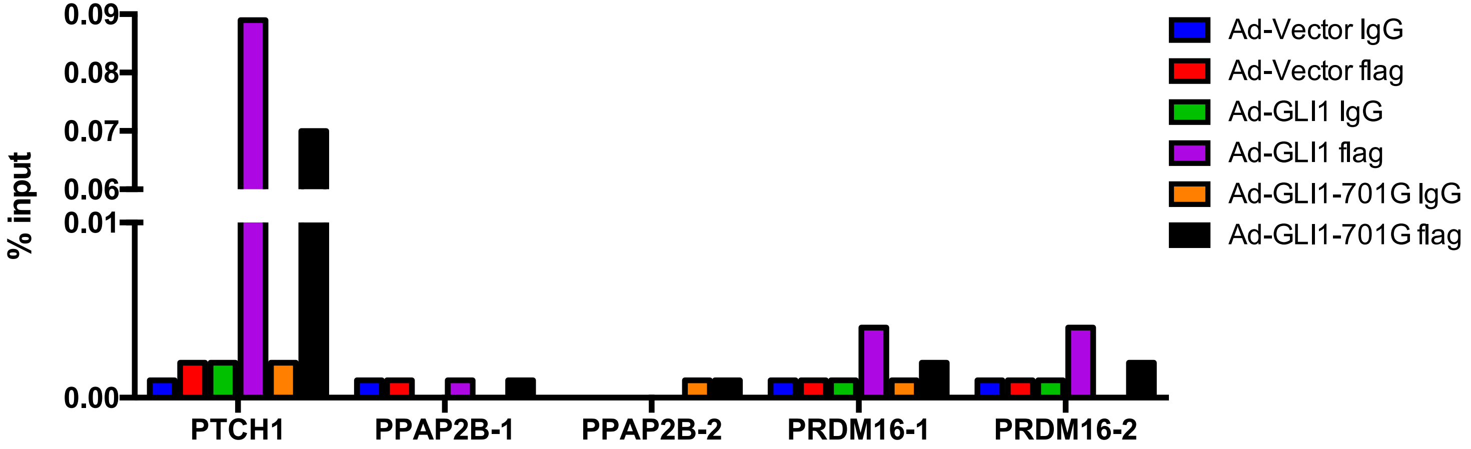

0.09
0.08
0.07
0.06
0.01
0.00
% input
PTCH1
PPAP2B-1
PPAP2B-2
PRDM16-1
PRDM16-2
Ad-Vector IgG
Ad-Vector flag
Ad-GLI1 IgG
Ad-GLI1 flag
Ad-GLI1-701G IgG
Ad-GLI1-701G flag

Supplementary Fig. S4

A

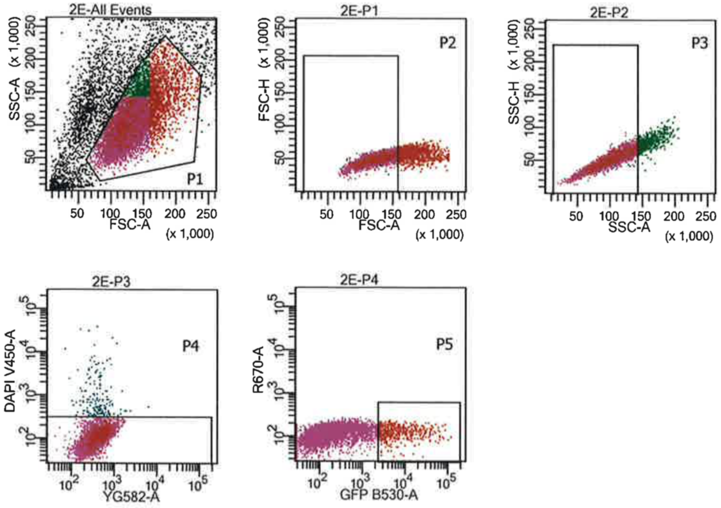

Tube: 2E

Population	#Events	%Parent	%Total
All Events	14,447	####	100.0
P1	10,765	74.5	74.5
P2	9,112	84.6	63.1
P3	8,498	93.3	58.8
P4	8,319	97.9	57.6
P5	482	5.8	3.3

B

```
3NE3-F    637  GTCTACTCACCACAGCCCCCCAGCATCACTGAGAATGCTGCCATGGAATGCTAGAGGGCT  696
               |||||||||||||||||||||||||||||||||||||||||||||| |||||||||||||
GLI1 WT  2128  GTCTACTCACCACAGCCCCCCAGCATCACTGAGAATGCTGCCATGG-ATGCTAGAGGGCT  2186

3NE3-R    142  CCACTGCCCACCATGGAGGTCCCAACTTCTGGCTCTTCCTGTAGCCCTCTAGCATTCCAT  201
               ||||||||||||||||||||||||||||||||||||||||||||||||||||||| ||||
GLI1 WT  2228  CCACTGCCCACCATGGAGGTCCCAACTTCTGGCTCTTCCTGTAGCCCTCTAGCAT-CCAT  2170
```

C

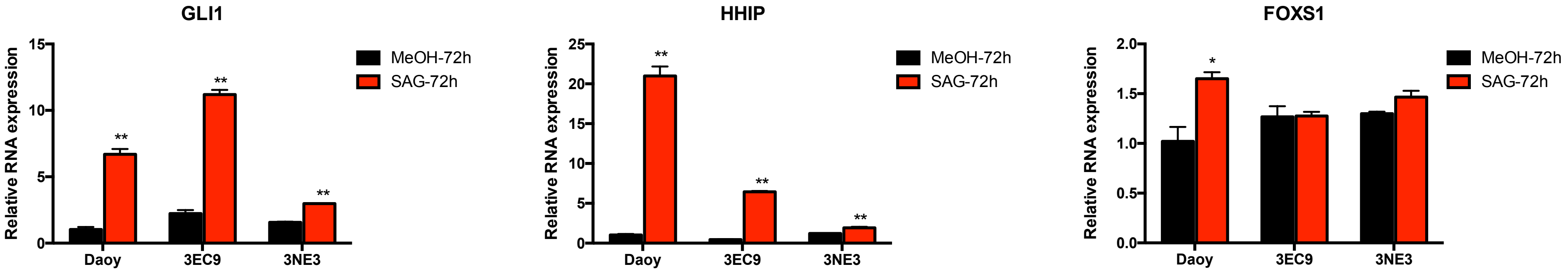
GLI1
HHIP
FOXS1
Relative RNA expression
MeOH-72h
SAG-72h
Daoy
3EC9
3NE3
**
*

D

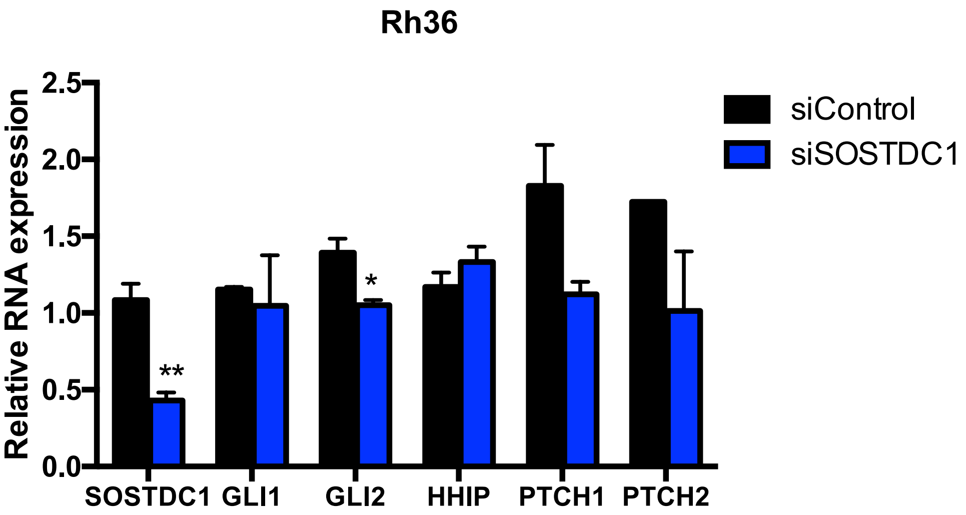
Rh36
Relative RNA expression
siControl
siSOSTDC1
SOSTDC1
GLI1
GLI2
HHIP
PTCH1
PTCH2
**
*

Supplementary Fig. S5

A

HEPM

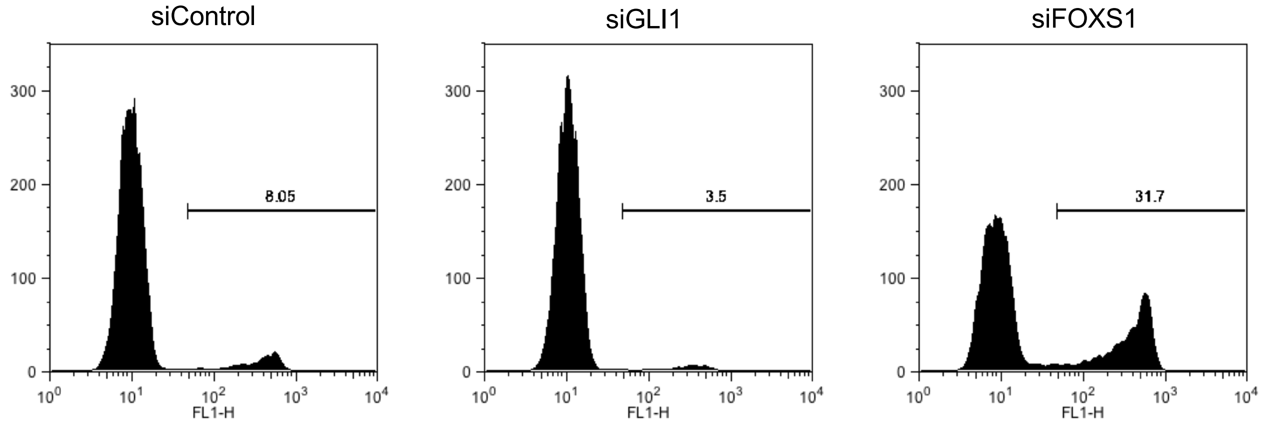

B

Rh36

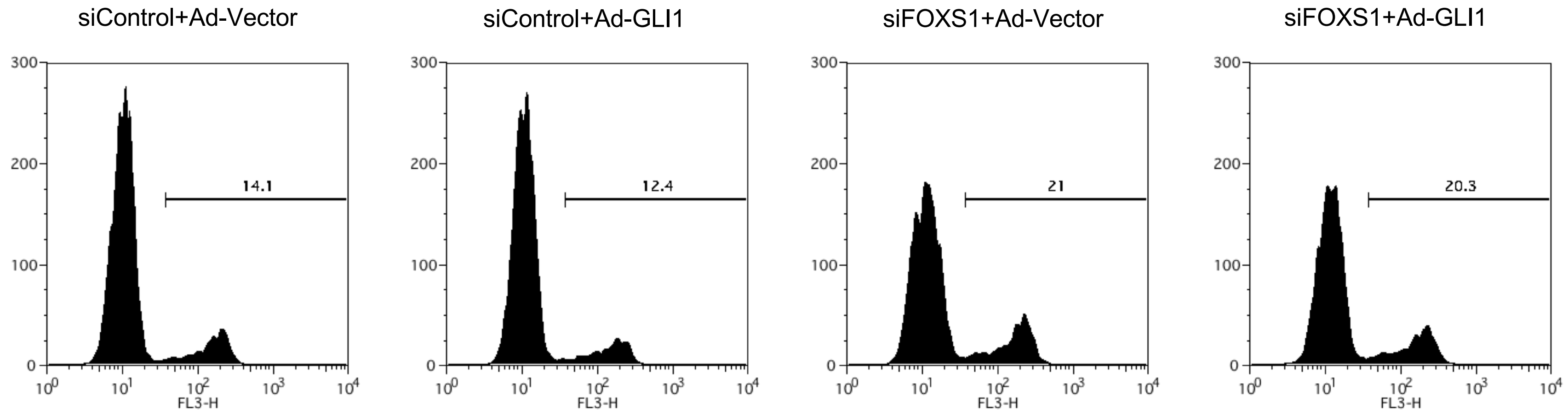

C

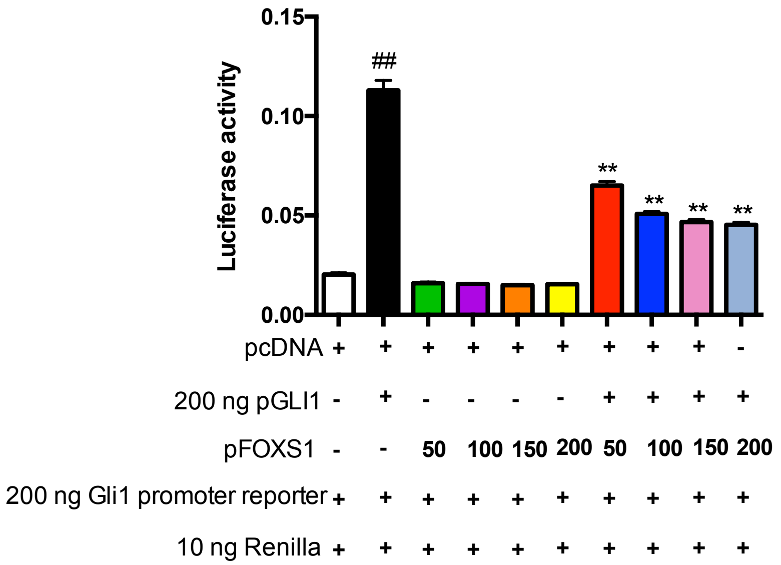

Luciferase activity
0.15
0.10
0.05
0.00
##
**
**
**
**
pcDNA + + + + + + + + + -
200 ng pGLI1 - + - - - - + + + +
pFOXS1 - - 50 100 150 200 50 100 150 200
200 ng Gli1 promoter reporter + + + + + + + + + +
10 ng Renilla + + + + + + + + + +

D

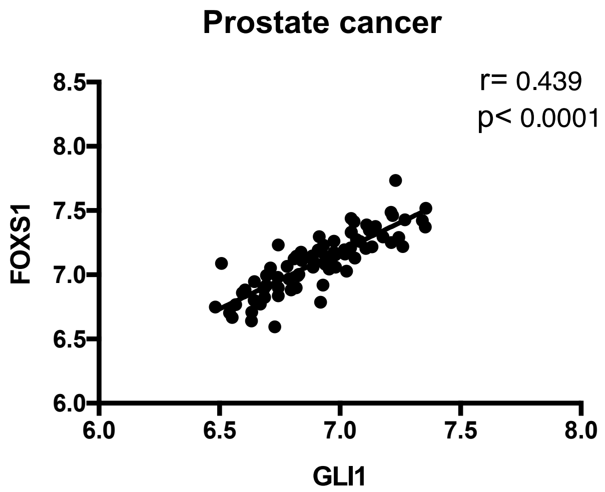

Prostate cancer
r= 0.439
p< 0.0001
FOXS1
8.5
8.0
7.5
7.0
6.5
6.0
6.0
6.5
7.0
7.5
8.0
GLI1
