# Supplementary material for: Identification of novel GLI1 target genes and regulatory circuits in human cancer cells
Source: Mol Oncol. 2018 Aug 30;12(10):1718–34. doi: 10.1002/1878-0261.12366 (PMC6166001; doi:10.1002/1878-0261.12366)
Supplement: Supplementary file 8 [file MOL2-12-1718-s008.pdf]

Supplementary figures

Supplementary Fig. S1. Genomic organization of *PTCH1* and *LOC100507346*.

A. The schematic figure showing the relative location of *PTCH1* (top) and *LOC100507346* (bottom). Exons are numbered and depicted as rectangles. Triangles indicate the transcription orientation. Red arrows marked on *LOC100507346* indicate the targeting regions of forward primer and reverse primer. **B.** Visualization of H3K27Ac marks according to the ENCODE data in the genomic region encompassing *LOC100507346*.

Supplementary Fig. S2. Heat map analysis of 29 randomly selected genes.

Heat map analysis of 29 randomly selected genes in both the knockdown (left) and the over-expression (right) datasets. The 4 lanes on the left panel indicate: biological replicates with GLI1 siRNAs (siGLI1-1 and siGLI1-2) and biological replicates with control siRNAs (siControl-1 and siControl-2). The 4 lanes on the right panel indicate: Rh36 cells over-expressing GLI1 (pGLI1), GLI1-701G (pGLI1-701G) and biological replicates of empty vector (pCMV-1 and pCMV-2). Red and blue colors indicate up- and down-regulation respectively, with the intensity highlighting the level of up- and down-regulation.

Supplementary Fig. S3. Validation of GLI1 target genes following over-expression of GLI1/GLI1-701G.

A. Western blot of HEK293A cells transfected with pAdTrack-CMV (pAd-Vector), pAdTrack-CMV-GLI1 (pAd-GLI1) and pAdTrack-CMV-GLI1-701G (pAd-GLI1-701G) plasmids. Both rabbit anti-GLI1 antibody (GLI1 Ab) and rabbit anti-flag antibody (Flag Ab) were used. Western blot of PTCH1, GLI1 and FOXS1 expression in Rh36 (**B**) and Daoy cells (**C**) transduced with two adenoviruses expressing GLI1 (Ad-GLI1: G11 and G44), two

adenoviruses expressing GLI1-701G (Ad-GLI1-701G: 17 and 19) or control adenoviruses (Ad-Vector). Rabbit anti-PTCH1 antibody, rabbit anti-flag antibody, mouse anti-β-actin and rabbit anti-FOXS1 antibody were used. *, indicates unspecific band. **D.** RNA expression of *SOX18* in Rh36 cells transfected with 0.5 μg or 1.0 μg plasmids of pCMV, pGLI1 and pGLI1-701G. Data from one representative experiment are shown as relative expression ($2^{-\Delta\Delta Ct}$ values). Error bars indicate standard deviation. **E.** RNA expression of *SOSTDC1* and *FOXS1* in Rh36 cells transfected with 0.5 μg, 1.0 μg, 2.0 μg or 4.0 μg plasmids of pCMV, pGLI1 and pGLI1-701G. Data from one representative experiment are shown as relative expression ($2^{-\Delta\Delta Ct}$ values). Error bars indicate standard deviation. **F.** ChIP-qPCR analyses of GLI1 binding on the promoter of the *PPAP2B* and the *PRDM16* genes in Daoy cells, following transduction with adenoviruses expressing GLI1 (Ad-GLI1), GLI1-701G (Ad-GLI1-701G) and control adenoviruses. Rabbit anti-flag antibody was used to detect GLI1, rabbit IgG was used as the control antibody. Two sets of primers on the *PPAR2B* promoter (PPAR2B-1 and PPAR2B-2) and two sets of primers on the *PRDM16* promoter (PRDM16-1 and PRDM16-2) were tested. The GLI1 binding on the *PTCH1* promoter served as a positive control. The data are normalized to input DNA.

Supplementary Fig. S4. The expression of FOXS1 in CRISPR/Cas9 mediated *GLI1* knockout Daoy subclones.

A. Daoy cells transfected with the CRISPR/Cas9 plasmid pSpCas9-GFP containing sgRNAs that target *GLI1* were sorted at single cell density into 96-well plates. Single cells were identified by sequential gating. Gate P1 indicates Daoy cell population; Gates P2 and P3 select singlets; Gate P4 indicates living cells and P5 gate indicates GFP positive Daoy cells. **B.** Genomic change in CRISPR/Cas9 mediated *GLI1* knockout Daoy subclone 3NE3. Comparison of the genomic sequence in the *GLI1* targeted region of the subclone to that of wild type *GLI1* (GLI1 WT), using forward (3NE3-F) (top) and reverse (3NE3-R) (bottom) primers. The single

nucleotide insertion in the subclone is highlighted in red. Nucleotide numbers in GLI1 WT refer to the GLI1 Genbank entry NM_005269. All four independent plasmids analyzed, harboring the GLI1 targeted region in 3NE3 cells, resulted in the same sequence. **C.** RNA expression of *GLI1*, *HHIP* and *FOXS1* in Daoy cells and CRISPR/Cas9 mediated *GLI1* knockout Daoy subclones (3EC9 and 3NE3) treated with 200 nM SAG or Methanol (MeOH) for 72 hours. Data from one representative experiment are shown as relative expression ($2^{-\Delta\Delta Ct}$ values). Error bars indicate standard deviation. Statistical significant, *P<0.05 and **P<0.01, compared to control, calculated by the Student's t-test. **D.** RNA expression of *GLI1*, *GLI2*, *HHIP*, *PTCH1* and *PTCH2* in Rh36 cells transfected with siRNAs targeting SOSTDC1 (siSOSTDC1) or control siRNAs (siControl). Data from biological triplicate experiments are shown as relative expression ($2^{-\Delta\Delta Ct}$ values). Error bars indicate standard error of the mean. Statistical significant, *P<0.05 and **P<0.01, compared to control, calculated by the Student's t-test.

Supplementary Fig. S5. FOXS1 regulates cell proliferation, inhibits GLI1 activity and correlates with GLI1 expression.

A. EdU incorporation assay of HEPM cells, cultured for 48 hours following siRNA transfection of siControl, siGLI1 and siFOXS1. Data from one representative experiment are shown in the histogram. **B.** EdU incorporation assay of Rh36 cells following transfection/transduction with siControl + Ad-Vector, siControl + Ad-GLI1, siFOXS1 + Ad-Vector or siFOXS1 + Ad-GLI1. Adenoviruses were added 6 hours after siRNA transfection. Data from one representative experiment are shown in the histogram. **C.** HEK293A cells were co-transfected with pcDNA3.1 vector (pcDNA), pCMV-GLI1-flag (pGLI1) or pcDNA-FOXS1 (pFOXS1), together with the mouse Gli1 promoter reporter plasmid and the control plasmid Renilla. Data from one representative experiment are shown. Error bars indicate standard deviation (SD). Statistical significant, $^{\#\#}$P < 0.01, compared to pcDNA; **P < 0.01, compared to pGLI1, calculated by the

Student's t-test. **D**. Scatter plot and Pearson correlation between *GLI1* and *FOXS1* expression in 72 prostate cancer samples.
